# Di-μ-oxido-bis­({2,2′-[ethane-1,2-diylbis(nitrilo­methanylyl­idene)]diphenolato}titanium(IV)) chloro­form disolvate

**DOI:** 10.1107/S1600536813029371

**Published:** 2013-10-31

**Authors:** Kirill V. Zaitsev, Sergey S. Karlov, Galina S. Zaitseva, Elmira Kh. Lermontova, Andrei V. Churakov

**Affiliations:** a Department of Chemistry, M.V. Lomonosov Moscow State University, Leninskie Gory 1/3, Moscow 119991, Russian Federation; b Institute of General and Inorganic Chemistry, Russian Academy of Sciences, Leninskii prosp. 31, Moscow 119991, Russian Federation

## Abstract

In the title compound, [Ti_2_(C_16_H_14_N_2_O_2_)_2_O_2_]·2CHCl_3_, the Ti^IV^ atom in the centrosymmetric complex has a distorted octa­hedral N_2_O_4_ coordination environment and is linked *via* two μ_2_-oxido bridges into a dinuclear centrosymmetric com­plex, with a Ti⋯Ti separation of 2.7794 (8) Å. In the salen (*N,N′*-ethyl­enebis(salicyl­imine)) ligand, the two salicyl­imine units make a dihedral angle of 45.31 (5)°. The complex mol­ecules are stacked parallel to [100], forming channels in which the solvent chloro­form mol­ecules are located. C—H⋯O hydrogen-bonding inter­actions between the complex mol­ecules and the solvent mol­ecules consolidate the crystal packing.

## Related literature

For general background to the chemistry of titanium complexes based on salen-type ligands, see: Gupta & Sutar (2008 ▶); Tsuchimoto (2001 ▶). For our previous work on titanium(IV) complexes with polydentate *N,O*-chelating ligands, see: Zaitsev *et al.* (2006 ▶, 2008 ▶).
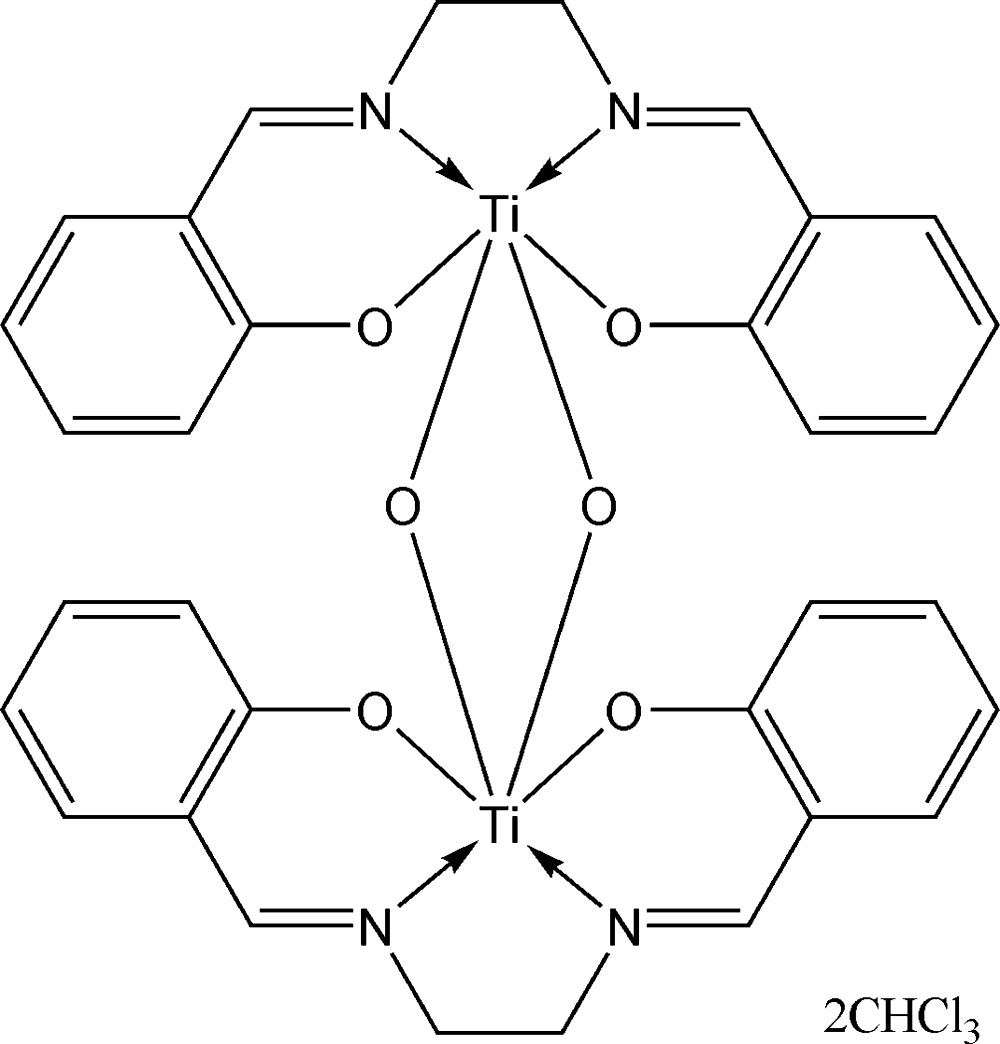


## Experimental

### 

#### Crystal data

[Ti_2_(C_16_H_14_N_2_O_2_)_2_O_2_]·2CHCl_3_
*M* *_r_* = 899.12
Monoclinic, 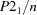

*a* = 8.8115 (10) Å
*b* = 11.4587 (13) Å
*c* = 18.785 (2) Å
β = 98.226 (2)°
*V* = 1877.2 (4) Å^3^
*Z* = 2
Mo *K*α radiation
μ = 0.90 mm^−1^
*T* = 150 K
0.25 × 0.08 × 0.06 mm

#### Data collection

Bruker APEXII CCD diffractometer
Absorption correction: multi-scan (*SADABS*; Bruker, 2008 ▶) *T*_min_ = 0.806, *T*_max_ = 0.948
16236 measured reflections
3677 independent reflections
3119 reflections with *I* > 2σ(*I*)
*R*_int_ = 0.028

#### Refinement

*R*[*F* ^2^ > 2σ(*F* ^2^)] = 0.031
*wR*(*F* ^2^) = 0.084
*S* = 1.04
3677 reflections
235 parameters
H-atom parameters constrained
Δρ_max_ = 0.47 e Å^−3^
Δρ_min_ = −0.28 e Å^−3^

### 

Data collection: *APEX2* (Bruker, 2008 ▶); cell refinement: *SAINT* (Bruker, 2008 ▶); data reduction: *SAINT*; program(s) used to solve structure: *SHELXTL* (Sheldrick, 2008 ▶); program(s) used to refine structure: *SHELXTL*; molecular graphics: *SHELXTL*; software used to prepare material for publication: *SHELXTL*.

## Supplementary Material

Crystal structure: contains datablock(s) I. DOI: 10.1107/S1600536813029371/wm2780sup1.cif

Structure factors: contains datablock(s) I. DOI: 10.1107/S1600536813029371/wm2780Isup2.hkl

Supplementary material file. DOI: 10.1107/S1600536813029371/wm2780Isup3.mol

Additional supplementary materials:  crystallographic information; 3D view; checkCIF report

## Figures and Tables

**Table 1:** Hydrogen-bond geometry (Å, °)

*D*—H⋯*A*	*D*—H	H⋯*A*	*D*⋯*A*	*D*—H⋯*A*
C1—H1⋯O1	1.00	2.08	3.029 (3)	157

**Fig. 1.**
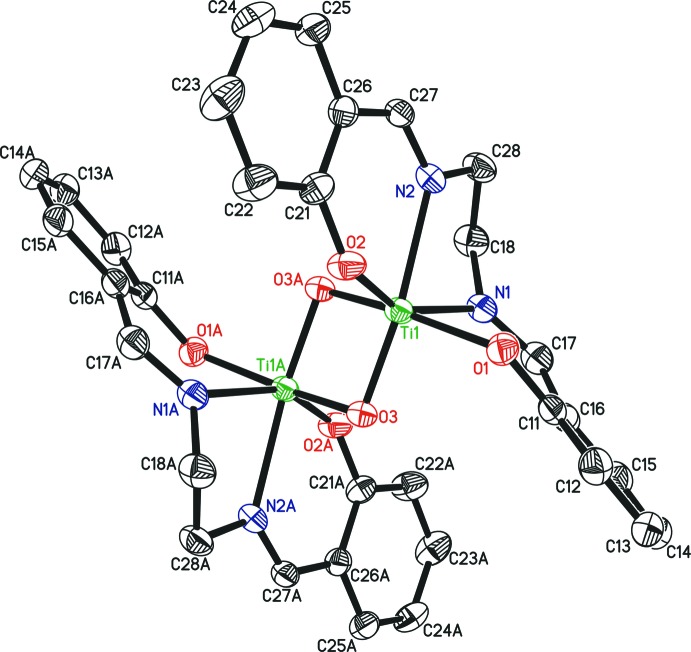
The molecular structure of the title compound, showing the numbering scheme. Displacement ellipsoids are drawn at the 50% probability level. Hydrogen atoms and the solvent molecules were omitted for clarity. [Symmetry code A) 1 - x, 2 - y, 1 - z.]

**Fig. 2.**
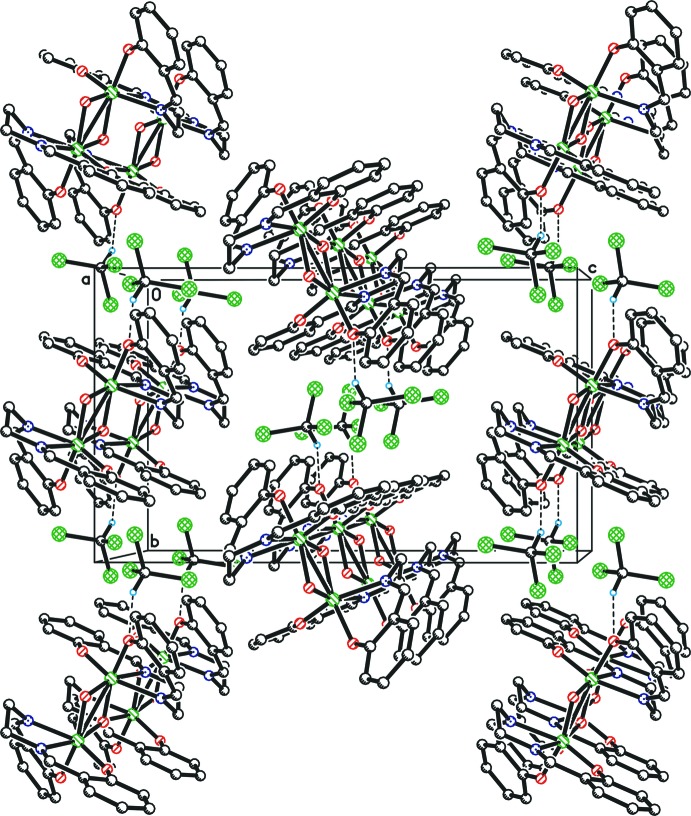
Channels extending parallel to [100], filled with chloroform solvent molecules. Cl3C—H···O hydrogen bonding interactions are shown as dashed lines.

## supplementary crystallographic information

### 1. Comment

As a part of our investigation on the chemistry of titanium complexes based on tridentate or tetradentate ligands (Zaitsev *et al.*, 2006, 2008) we obtained and studied the structure of the title compound, [Ti(O)(C_16_H_14_N_2_O_2_)]_2_ (or [Ti(O)(salen)]_2_) that crystallizes with two chloroform solvent molecules. For general background to the chemistry of titanium complexes based on salen-type ligands, see: Gupta & Sutar (2008).

The title salen complex is centrosymmetric. The Ti(IV) atoms are linked by µ_2_-oxido bridges and possess a distorted octahedral N_2_O_4_ coordination environment with *cis* interligand angles ranging from 82.20 (6) to 106.51 (7) °. In the central Ti_2_(µ_2_-O)_2_ fragment, the metal–oxygen distances are significantly different (1.8029 (14) and 1.9029 (15) Å). The opposite N–Ti bond lengths also vary by approximately 0.1 Å (2.1502 (17) and 2.2555 (17) Å). The same structural feature was previously reported for another solvatomorph of this complex (Tsuchimoto, 2001). In the ligand, the two salicylimine fragmets form a dihedral angle of 45.31 (5) ° (Fig. 1).

In the crystal, solvent chloroform molecule are linked *via* C—H···O hydrogen bonding interactions with the main molecule (Table 1). The solvent molecules fill channels spreading parallel to [100] (Fig. 2). The adjacent titanium complexes are connected by T-shaped C—H···π interactions. However, no π···π- stacking interactions are observed in this structure.

### 2. Experimental

The title compound was obtained from reaction of equimolar amounts of Ti(O-*i*Pr)_4_ and salen in chloroform as a solid which is insoluble in common organic solvents. The crystals suitable for X-Ray analysis crystallized from the reaction mixture.

### 3. Refinement

All hydrogen atoms were placed in calculated positions and refined using a riding model with C—H = 1.00 Å and *U*_iso_(H) = 1.2*U*_eq_(C) for chloroform molecule; C—H = 0.99 Å and *U*_iso_(H) = 1.2*U*_eq_(C) for methylene groups; C—H = 0.95 Å and *U*_iso_(H) = 1.2*U*_eq_(C) for *sp*^2^ carbon atoms.

### Crystal data

**Table:**

[Ti_2_(C_16_H_14_N_2_O_2_)_2_O_2_]·2CHCl_3_	*F*(000) = 912
*M**_r_* = 899.12	*D*_x_ = 1.591 Mg m^−^^3^
Monoclinic, *P*2_1_/*n*	Mo *K*α radiation, λ = 0.71073 Å
Hall symbol: -P 2yn	Cell parameters from 5413 reflections
*a* = 8.8115 (10) Å	θ = 2.4–29.6°
*b* = 11.4587 (13) Å	µ = 0.90 mm^−^^1^
*c* = 18.785 (2) Å	*T* = 150 K
β = 98.226 (2)°	Block, colourless
*V* = 1877.2 (4) Å^3^	0.25 × 0.08 × 0.06 mm
*Z* = 2	

### Data collection

**Table:**

Bruker APEXII CCD diffractometer	3677 independent reflections
Radiation source: fine-focus sealed tube	3119 reflections with *I* > 2σ(*I*)
Graphite monochromator	*R*_int_ = 0.028
ω scans	θ_max_ = 26.0°, θ_min_ = 2.1°
Absorption correction: multi-scan (*SADABS*; Bruker, 2008)	*h* = −10→10
*T*_min_ = 0.806, *T*_max_ = 0.948	*k* = −14→14
16236 measured reflections	*l* = −22→23

### Refinement

**Table:**

Refinement on *F*^2^	Primary atom site location: structure-invariant direct methods
Least-squares matrix: full	Secondary atom site location: difference Fourier map
*R*[*F*^2^ > 2σ(*F*^2^)] = 0.031	Hydrogen site location: inferred from neighbouring sites
*wR*(*F*^2^) = 0.084	H-atom parameters constrained
*S* = 1.04	*w* = 1/[σ^2^(*F*_o_^2^) + (0.0415*P*)^2^ + 1.1985*P*] where *P* = (*F*_o_^2^ + 2*F*_c_^2^)/3
3677 reflections	(Δ/σ)_max_ = 0.001
235 parameters	Δρ_max_ = 0.47 e Å^−^^3^
0 restraints	Δρ_min_ = −0.28 e Å^−^^3^

### Special details

**Table:**

Geometry. All e.s.d.'s (except the e.s.d. in the dihedral angle between two l.s. planes) are estimated using the full covariance matrix. The cell e.s.d.'s are taken into account individually in the estimation of e.s.d.'s in distances, angles and torsion angles; correlations between e.s.d.'s in cell parameters are only used when they are defined by crystal symmetry. An approximate (isotropic) treatment of cell e.s.d.'s is used for estimating e.s.d.'s involving l.s. planes.
Refinement. Refinement of *F*^2^ against ALL reflections. The weighted *R*-factor *wR* and goodness of fit *S* are based on *F*^2^, conventional *R*-factors *R* are based on *F*, with *F* set to zero for negative *F*^2^. The threshold expression of *F*^2^ > σ(*F*^2^) is used only for calculating *R*-factors(gt) *etc*. and is not relevant to the choice of reflections for refinement. *R*-factors based on *F*^2^ are statistically about twice as large as those based on *F*, and *R*- factors based on ALL data will be even larger.

### Fractional atomic coordinates and isotropic or equivalent isotropic displacement parameters (Å^2^)

**Table:**

	*x*	*y*	*z*	*U*_iso_*/*U*_eq_	
Ti1	0.55329 (4)	0.89882 (3)	0.471151 (19)	0.02192 (11)	
O3	0.38110 (15)	0.94635 (12)	0.50409 (7)	0.0250 (3)	
O1	0.44879 (16)	0.76247 (12)	0.42307 (7)	0.0266 (3)	
O2	0.66652 (16)	0.81354 (14)	0.54587 (8)	0.0292 (3)	
N1	0.50499 (19)	0.96203 (15)	0.36249 (9)	0.0256 (4)	
N2	0.77616 (19)	0.89426 (14)	0.42525 (9)	0.0241 (4)	
C11	0.3151 (2)	0.76229 (18)	0.38023 (11)	0.0255 (4)	
C12	0.2143 (3)	0.6677 (2)	0.38226 (12)	0.0329 (5)	
H12	0.2425	0.6046	0.4142	0.039*	
C13	0.0748 (3)	0.6657 (2)	0.33826 (13)	0.0374 (6)	
H13	0.0062	0.6025	0.3413	0.045*	
C14	0.0334 (3)	0.7561 (2)	0.28908 (12)	0.0362 (5)	
H14	−0.0625	0.7540	0.2587	0.043*	
C15	0.1313 (2)	0.8470 (2)	0.28506 (12)	0.0328 (5)	
H15	0.1036	0.9074	0.2510	0.039*	
C16	0.2732 (2)	0.85321 (19)	0.33048 (11)	0.0274 (5)	
C17	0.3830 (2)	0.94051 (19)	0.31813 (12)	0.0299 (5)	
H17	0.3647	0.9847	0.2750	0.036*	
C18	0.6317 (3)	1.0275 (2)	0.34028 (12)	0.0317 (5)	
H18A	0.6505	1.0999	0.3690	0.038*	
H18B	0.6091	1.0487	0.2888	0.038*	
C21	0.8128 (2)	0.79353 (18)	0.57183 (11)	0.0267 (4)	
C22	0.8487 (3)	0.7483 (2)	0.64094 (13)	0.0370 (6)	
H22	0.7693	0.7355	0.6693	0.044*	
C23	0.9989 (3)	0.7218 (2)	0.66894 (13)	0.0399 (6)	
H23	1.0214	0.6916	0.7164	0.048*	
C24	1.1163 (3)	0.7389 (2)	0.62846 (13)	0.0360 (5)	
H24	1.2185	0.7180	0.6472	0.043*	
C25	1.0835 (2)	0.78687 (19)	0.56034 (12)	0.0311 (5)	
H25	1.1645	0.8006	0.5330	0.037*	
C26	0.9329 (2)	0.81553 (18)	0.53108 (11)	0.0248 (4)	
C27	0.9073 (2)	0.86562 (18)	0.45940 (11)	0.0251 (4)	
H27	0.9946	0.8781	0.4360	0.030*	
C28	0.7706 (2)	0.9466 (2)	0.35350 (11)	0.0303 (5)	
H28A	0.7620	0.8846	0.3164	0.036*	
H28B	0.8657	0.9913	0.3507	0.036*	
C1	0.6290 (3)	0.54038 (19)	0.40993 (12)	0.0301 (5)	
H1	0.5673	0.6049	0.4273	0.036*	
Cl1	0.81195 (7)	0.54170 (5)	0.46134 (3)	0.04022 (16)	
Cl2	0.53362 (6)	0.40740 (5)	0.42008 (3)	0.03615 (15)	
Cl3	0.64366 (8)	0.56598 (6)	0.31832 (3)	0.04712 (18)	

### Atomic displacement parameters (Å^2^)

**Table:**

	*U*^11^	*U*^22^	*U*^33^	*U*^12^	*U*^13^	*U*^23^
Ti1	0.01996 (19)	0.0259 (2)	0.02059 (19)	0.00184 (14)	0.00518 (14)	0.00209 (14)
O3	0.0203 (7)	0.0296 (8)	0.0263 (8)	−0.0016 (6)	0.0078 (6)	−0.0015 (6)
O1	0.0258 (7)	0.0272 (8)	0.0259 (8)	0.0027 (6)	0.0008 (6)	0.0007 (6)
O2	0.0211 (7)	0.0387 (9)	0.0284 (8)	0.0015 (6)	0.0051 (6)	0.0092 (6)
N1	0.0260 (9)	0.0274 (9)	0.0241 (9)	0.0040 (7)	0.0056 (7)	0.0017 (7)
N2	0.0263 (9)	0.0240 (9)	0.0231 (9)	0.0030 (7)	0.0078 (7)	0.0001 (7)
C11	0.0239 (10)	0.0294 (11)	0.0234 (10)	0.0043 (8)	0.0040 (8)	−0.0058 (8)
C12	0.0357 (12)	0.0347 (12)	0.0282 (12)	−0.0003 (10)	0.0047 (10)	−0.0015 (9)
C13	0.0327 (12)	0.0462 (14)	0.0341 (13)	−0.0095 (11)	0.0074 (10)	−0.0124 (11)
C14	0.0254 (11)	0.0503 (15)	0.0311 (12)	0.0020 (10)	−0.0017 (9)	−0.0127 (11)
C15	0.0306 (12)	0.0409 (13)	0.0259 (11)	0.0104 (10)	0.0012 (9)	−0.0045 (10)
C16	0.0269 (11)	0.0331 (11)	0.0226 (10)	0.0058 (9)	0.0047 (8)	−0.0036 (9)
C17	0.0322 (12)	0.0334 (12)	0.0238 (11)	0.0072 (9)	0.0031 (9)	0.0042 (9)
C18	0.0347 (12)	0.0344 (12)	0.0273 (11)	0.0004 (10)	0.0087 (9)	0.0088 (9)
C21	0.0239 (10)	0.0249 (11)	0.0304 (11)	−0.0028 (8)	0.0014 (9)	0.0023 (8)
C22	0.0295 (12)	0.0474 (14)	0.0341 (13)	−0.0033 (10)	0.0046 (10)	0.0141 (10)
C23	0.0377 (13)	0.0441 (14)	0.0350 (13)	−0.0075 (11)	−0.0051 (10)	0.0141 (11)
C24	0.0240 (11)	0.0355 (13)	0.0453 (14)	−0.0017 (9)	−0.0059 (10)	0.0028 (10)
C25	0.0240 (11)	0.0334 (12)	0.0351 (12)	−0.0017 (9)	0.0018 (9)	−0.0044 (10)
C26	0.0246 (10)	0.0223 (10)	0.0275 (11)	−0.0019 (8)	0.0038 (8)	−0.0032 (8)
C27	0.0229 (10)	0.0260 (10)	0.0276 (11)	−0.0009 (8)	0.0078 (8)	−0.0054 (8)
C28	0.0296 (11)	0.0376 (12)	0.0260 (11)	0.0025 (9)	0.0115 (9)	0.0028 (9)
C1	0.0340 (12)	0.0290 (11)	0.0280 (11)	−0.0003 (9)	0.0070 (9)	0.0014 (9)
Cl1	0.0384 (3)	0.0407 (3)	0.0393 (3)	−0.0079 (3)	−0.0021 (3)	0.0001 (2)
Cl2	0.0354 (3)	0.0328 (3)	0.0417 (3)	−0.0068 (2)	0.0105 (2)	−0.0009 (2)
Cl3	0.0518 (4)	0.0621 (4)	0.0276 (3)	−0.0147 (3)	0.0063 (3)	0.0046 (3)

### Geometric parameters (Å, º)

**Table:**

Ti1—O3	1.8029 (14)	C16—C17	1.433 (3)
Ti1—O2	1.8760 (15)	C17—H17	0.9500
Ti1—O3^i^	1.9029 (15)	C18—C28	1.527 (3)
Ti1—O1	1.9664 (15)	C18—H18A	0.9900
Ti1—N1	2.1502 (17)	C18—H18B	0.9900
Ti1—N2	2.2555 (17)	C21—C22	1.391 (3)
Ti1—Ti1^i^	2.7794 (8)	C21—C26	1.415 (3)
O3—Ti1^i^	1.9029 (15)	C22—C23	1.386 (3)
O1—C11	1.328 (2)	C22—H22	0.9500
O2—C21	1.331 (2)	C23—C24	1.382 (3)
N1—C17	1.287 (3)	C23—H23	0.9500
N1—C18	1.455 (3)	C24—C25	1.384 (3)
N2—C27	1.282 (3)	C24—H24	0.9500
N2—C28	1.470 (3)	C25—C26	1.401 (3)
C11—C12	1.405 (3)	C25—H25	0.9500
C11—C16	1.413 (3)	C26—C27	1.451 (3)
C12—C13	1.380 (3)	C27—H27	0.9500
C12—H12	0.9500	C28—H28A	0.9900
C13—C14	1.401 (4)	C28—H28B	0.9900
C13—H13	0.9500	C1—Cl1	1.756 (2)
C14—C15	1.361 (3)	C1—Cl2	1.764 (2)
C14—H14	0.9500	C1—Cl3	1.768 (2)
C15—C16	1.411 (3)	C1—H1	1.0000
C15—H15	0.9500		
			
O3—Ti1—O2	106.51 (7)	N1—C17—C16	123.4 (2)
O3—Ti1—O3^i^	82.86 (6)	N1—C17—H17	118.3
O2—Ti1—O3^i^	101.09 (7)	C16—C17—H17	118.3
O3—Ti1—O1	92.06 (6)	N1—C18—C28	105.69 (17)
O2—Ti1—O1	95.38 (6)	N1—C18—H18A	110.6
O3^i^—Ti1—O1	163.53 (6)	C28—C18—H18A	110.6
O3—Ti1—N1	99.33 (7)	N1—C18—H18B	110.6
O2—Ti1—N1	153.83 (7)	C28—C18—H18B	110.6
O3^i^—Ti1—N1	85.98 (6)	H18A—C18—H18B	108.7
O1—Ti1—N1	79.37 (6)	O2—C21—C22	119.04 (19)
O3—Ti1—N2	163.69 (6)	O2—C21—C26	122.08 (19)
O2—Ti1—N2	82.82 (6)	C22—C21—C26	118.87 (19)
O3^i^—Ti1—N2	82.20 (6)	C23—C22—C21	120.9 (2)
O1—Ti1—N2	100.49 (6)	C23—C22—H22	119.6
N1—Ti1—N2	73.13 (6)	C21—C22—H22	119.6
Ti1—O3—Ti1^i^	97.15 (6)	C24—C23—C22	120.6 (2)
C11—O1—Ti1	126.58 (13)	C24—C23—H23	119.7
C21—O2—Ti1	138.36 (13)	C22—C23—H23	119.7
C17—N1—C18	121.17 (18)	C23—C24—C25	119.4 (2)
C17—N1—Ti1	125.73 (15)	C23—C24—H24	120.3
C18—N1—Ti1	112.97 (13)	C25—C24—H24	120.3
C27—N2—C28	118.22 (18)	C24—C25—C26	121.1 (2)
C27—N2—Ti1	125.92 (14)	C24—C25—H25	119.5
C28—N2—Ti1	115.12 (13)	C26—C25—H25	119.5
O1—C11—C12	119.60 (19)	C25—C26—C21	119.08 (19)
O1—C11—C16	121.65 (19)	C25—C26—C27	117.97 (19)
C12—C11—C16	118.72 (19)	C21—C26—C27	122.94 (18)
C13—C12—C11	120.6 (2)	N2—C27—C26	125.07 (19)
C13—C12—H12	119.7	N2—C27—H27	117.5
C11—C12—H12	119.7	C26—C27—H27	117.5
C12—C13—C14	120.5 (2)	N2—C28—C18	108.51 (17)
C12—C13—H13	119.7	N2—C28—H28A	110.0
C14—C13—H13	119.7	C18—C28—H28A	110.0
C15—C14—C13	119.7 (2)	N2—C28—H28B	110.0
C15—C14—H14	120.2	C18—C28—H28B	110.0
C13—C14—H14	120.2	H28A—C28—H28B	108.4
C14—C15—C16	121.3 (2)	Cl1—C1—Cl2	111.23 (12)
C14—C15—H15	119.4	Cl1—C1—Cl3	110.17 (12)
C16—C15—H15	119.4	Cl2—C1—Cl3	110.53 (12)
C15—C16—C11	119.2 (2)	Cl1—C1—H1	108.3
C15—C16—C17	119.8 (2)	Cl2—C1—H1	108.3
C11—C16—C17	120.21 (19)	Cl3—C1—H1	108.3

Symmetry code: (i) −*x*+1, −*y*+2, −*z*+1.

### Hydrogen-bond geometry (Å, º)

**Table:**

*D*—H···*A*	*D*—H	H···*A*	*D*···*A*	*D*—H···*A*
C1—H1···O1	1.00	2.08	3.029 (3)	157
